# Prognostic Value of EZH2 Expression and Activity in Renal Cell Carcinoma: A Prospective Study

**DOI:** 10.1371/journal.pone.0081484

**Published:** 2013-11-27

**Authors:** Li Liu, Zhibing Xu, Lei Zhong, Hang Wang, Shuai Jiang, Qilai Long, Jiejie Xu, Jianming Guo

**Affiliations:** 1 Department of Urology, Zhongshan Hospital, Fudan University, Shanghai, People’s Republic of China; 2 Department of Biochemistry and Molecular Biology, School of Basic Medical Sciences, Shanghai Medical College of Fudan University, Shanghai, China; Vanderbilt University Medical Center, United States of America

## Abstract

Increased expression of EZH2 correlates with aggressive clinical behavior in various malignancies. In this study, we aim to investigate the clinical and prognostic values of EZH2 expression and activity in tumor tissues and improve the risk stratification in patients with renal cell carcinoma after surgery. We analyzed EZH2 expression and its activity as indicated by H3K27me3 levels comprising 373 patients with renal cell carcinoma in our institute. Outcome was assessed as overall survival and disease free survival using Kaplan-Meier analysis. Prognostic values of EZH2 and H3K27me3 expression for clinical outcomes were evaluated by Cox regression analysis. We used receiver operating characteristic to calculate diagnostic accuracy. High EZH2 expression correlates with poor overall survival in all patients, especially in advanced RCC, which is an independent prognostic factor in disease free survival and overall survival. Compared with EZH2, H3K27me3 expression is not an independent prognostic factor. The expressions of H3K27me3 and EZH2 are not completely consistent, which might be due to complicated interaction of Polycomb Repressor Complex 2. A combination of EZH2 expression and TNM stage could have better prognostic value than do TNM stage or EZH2 expression alone in both sets for disease free survival and overall survival. These results imply that evaluating intratumoral EZH2 density might improve prognostic value to the TNM staging system and inform treatment decisions for patients with late-stage renal cell carcinoma.

## Introduction

Kidney and renal pelvis cancer were estimated to account for 65,150 new cases and 13,680 cancer-related deaths in the USA in 2013 [1]. About 25% of renal cell carcinoma (RCC) patients diagnosed with localized disease who undergo surgery subsequently experience recurrence of the disease [2]. The current predictive risk factors of outcome include TNM stage, tumor size, and Fuhrman grade [3]. However, patients with similar clinical stages have various outcomes. Although, VHL mutation leads to activated HIF signaling in clear cell renal cell carcinoma (ccRCC). It still cannot interpretate well why the tumor account for the different behaviors. It is still unclear why RCC have the different biological behaviors. A recent study suggested that exploration of epigenetic modification might indicate significant biological properties and offer clues to novel therapy for RCC [4].

Histone modification is a crucial epigenetic modification. Recent findings suggested that SETD2, EZH2, and MLL2 methyltransferases, and UTX and JARID1C demethylases played crucial roles in the development of RCC [5,6]. However, whether any of these modifications affect the outcome and how they work are still unknown in RCC patients. EZH2 is the catalytic core protein in the Polycomb Repressor Complex 2 (PRC2), which is an important family of Polycomb Group (PcG) chromatin-modifying complexes. PcG proteins are transcriptional repressors. Transcriptional dysregulation of cells can lead to serious developmental defects [7,8]. As a histone methyltransferase (HMT), EZH2 catalyzes the trimethylation of lysine 27 of histone H3 (H3K27me3). It can silence tumor suppress genes which play a role in cell cycle regulation, senescence, cell fate decision, and cell differentiation [9,10]. EZH2 disturbance could be a pivotal driver of tumor development.

In recent studies, overexpression of EZH2 is related to poor prognostic in cancers of the prostate, breast, melanoma, endometrium, and pancreas [11-13]. However, the prognostic value and risk stratification significance of EZH2 in RCC remains far from full understand in large patient cohorts. Our aim is to examine the expression of EZH2 and H3K27me3 in RCC patients, explore their clinical significance in disease progression and assess the prognostic value of EZH2 as a tissue protein marker for RCC.

## Materials and Methods

### Ethics statement

Ethical approval was granted by the Clinical Research Ethics Committee of Zhongshan Hospital of Fudan University (Shanghai, China). Signed informed consent was obtained from all patients for the acquisition and use of patient tissue samples and anonymized clinical data.

### Patients and eligibility criteria

We enrolled patients from the Department of Urology, Zhongshan Hospital, Fudan University, and whose tumors were pathologically proven RCC. This database, started in 2005, is designed to prospectively study RCC patients undergoing nephrectomy. It includes patients’ baseline clinical and outcome data. Fuhrman grading system and TNM stage were according to 2004 WHO criteria and the American Joint Committee on Cancer 2010 TNM classification respectively [14-16]. The first patient was enrolled on Feb 8, 2005, and last patient enrolled on Jun 30, 2007. Patients were excluded if samples were necrotic and hemorrhagic in large area (39) or follow-up information was missing (57). None of the patients had received any adjuvant therapy before operation. All patients were followed up prospectively by the treating physician every three months. Metastasis and recurrence were defined on the basis of ultrasound and CT characteristics or were confirmed by histopathology. On the basis of a computer-generated allocation sequence, we randomly divided the 373 specimens into a 187 sample training set and a 186 sample validation set.

### Immunohistochemistry and evaluation

We analyzed the EZH2 and H3K27me3 expression by immunohistochemistry. The immunohistochemical procedure (IHC protocol F) was performed on an automated immunostainer (LEICA CO.LTD, Germany). Primary antibodies against EZH2 (1:100 dilution; Cell Signaling Technology, Danvers, MA) and H3K27me3 (1:2000 dilution; Millipore, Billerica, MA) were used in the procedure. Two observers (Shuai. J and Lei. Z), unaware of the clinical features and outcomes of patients, evaluated slides. As previously described, scores were calculated by multiplying the staining intensity and extension ranging from 0 to 12 for each tumor or non-tumor tissue score [17]. Briefly, category A documented the intensity of IHC staining as 0 (negative), 1 (weak), 2 (moderate), and 3 (strong). Category B documented the percentage of immunoreactive cell as 1 (0-25%), 2 (26-50%), 3 (51-75%), and 4 (76-100%). Multiplication of category A and B resulted in an IRS ranging from 0 to 12 for each specimen. We obtained the optimum cutoff value of score by receiver-operator characteristic (ROC) analysis, according to 5-year overall survival (OS). We designated low expression as total score 0-1 and high expression as 2-12 for EZH2, and low expression as total score 0-3 and high expression as 4-12 for H3K27me3. 

### Western blot

Western blot was carried out as previously described [18]. Primary antibodies used in Western blot included those against EZH2 (Cell Signaling Technology), H3K27me3 (Millipore), H3 (Abcam, Cambridge, MA) and GAPDH (Santa Cruz Biotechnology, Santa Cruz, CA).

### Survival analysis

DFS is defined as the interval between curative surgery and recurrence or metastasis. OS is defined as the interval from date of curative surgery until death from any cause. The patients were cencerd if they were still alive until the last follow-up. We assessed disease-free survival and overall survival using Kaplan-Meier method and log-rank test.

### Statistical analysis

We analyzed associations between EZH2 or H3K27me3 expression and clinical characteristics with χ^2^ test, Fisher’s exact method, or CMH (Cochran-Mantel-Haenszel) χ^2^. Cox univariate analysis was performed. Those parameters with a p-value< 0.1 in the univariate analyses were included in a Cox multivariate proportional hazards regression model. We used ROC curves to assess sensitivity, specificity and respective areas under the curves (AUCs) with 95% (CI) for the prediction of survival. We used Logistic stepwise regression to estimate the EZH2, H3K27me3 and TNM diagnostic accuracy. The variable was removed if the p-value was greater than 0.05. A new variable predicted probability (p) for RCC was obtained by Logistic stepwise regression on the basis of an equation. All equations were provided in the table S1. All p-values were two sided, and differences were considered significant at values of p< 0.05. Statistical analysis was carried out using GraphPad Prism 5 and Stata 11.0.

## Results

### Patient characteristics and immunohistochemical findings

**Figure 1:**
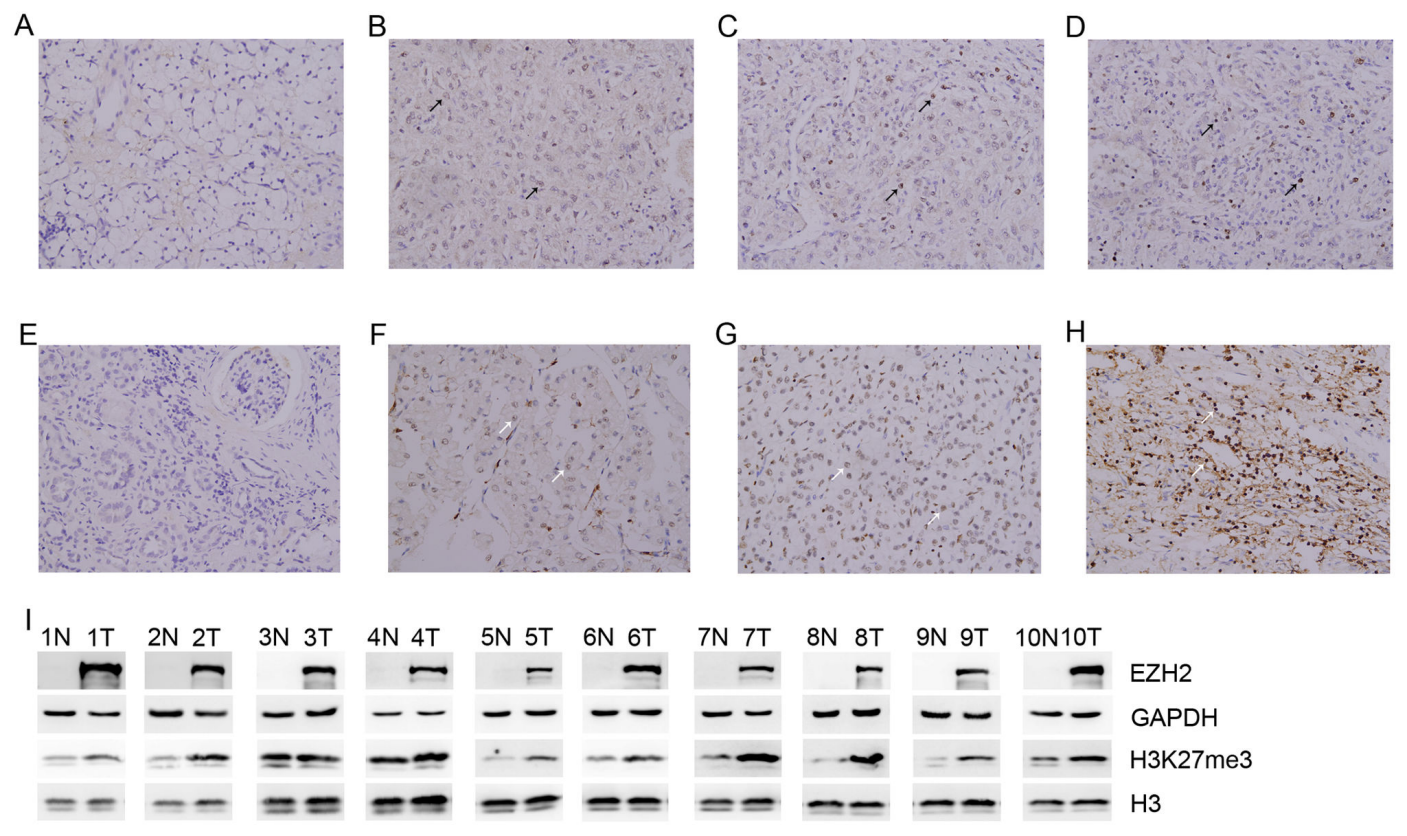
The expression of EZH2 and H3K27me3 in human samples. Representative EZH2 and H3K27me3 immunohistochemical staining in renal cell carcinoma (200×magnification). (A-H) Positive EZH2 (black arrow) and H3K27me3 (white arrow) display a nuclear staining. (A) negative in RCC (E) negative in none tumor tissue (B-D) EZH2 positive (F-H) H3K27me3 positive (B) (F) staining intensity index-1socre (C) (G) staining intensity index-2 score (D) (H) staining intensity index-3 score (I) the expression of EZH2 and H3K27me3 was detected in all 10 cases of RCC tissues compared to adjacent non-RCC tissues. N, non-RCC tissue; T, RCC tissue.

A total of 373 patients with RCC from two sets were included in this study. Clinical characteristics were much the same in each cohort. Most of the patients were men, and had clear-cell histology, Fuhrman grade II, and TNM stage I or II diseases. The median follow-up was 77 months (IQR 72-83) in the training set and 75 months (IQR 72- 82) in the validation set (table 1). Representative examples were showed in Figure 1. EZH2 and H3K27me3 immunostaining were mainly located in the nuclei of RCC. Negative staining and positive staining with IHC intensities 1, 2, and 3 of the tumor tissue are shown in Figure 1.EZH2 staining is absent in non-tumorous renal tissues (Figure 1 e). According to the criterion, 46.5% and 55.4% cases had low EZH2 expression in the training and validation sets, respectively. 51.3% and 54.3% patients were scored low H3K27me3 expression in the training and validation sets, respectively (table S2) (table S3). EZH2 expression is significantly associated with TNM stage in training and validation sets (p=0.012 and p=0.002, respectively). Additionally, EZH2 associates with T classification (p=0.011) in validation set. H3K27me3 expression is significantly associated with Fuhrman grade, T classification, and TNM stage (p=0.047, p=0.008, and p=0.003, respectively) in validation set, respectively. In training set, there are no statistically significant correlations between H3K27me3 expression and clinical characteristics (table S2) (table S3). 

**Table 1:** Patient characteristics of two independent RCC sets.

**Characteristic**	**Training set (n=187)**	**Validation set (n=186)**
Follow-up (months)	77 (72-83)	75 (72-82)
Age (years)	55 (45-62)	54 (46-63)
Sex		
Female	52 (27.8%)	62 (33.3%)
Male	135 (72.2%)	124 (66.7%)
Histology		
Clear cell	173 (92.5%)	169 (90.9%)
Papillary	10 (5.3%)	6 (3.2%)
Chromophobe	2 (1.1%)	8 (4.3%)
Others	2 (1.1%)	3 (1.6%)
ECOG PS		
0	142 (75.9%)	144 (77.4%)
≥1	45 (24.1%)	42 (22.6%)
Fuhrman nuclear grade (ccRCC)		
1	29 (15.5%)	21 (11.3%)
2	133 (71.1%)	132 (70.9%)
3	22 (11.8%)	31 (16.7%)
4	3 (1.6%)	2 (1.1%)
pT		
T1	126 (67.4%)	115 (61.8%)
T2	12 (6.4%)	19 (10.2%)
T3	48 (25.7%)	49 (26.4%)
T4	1 (0.5%)	3 (1.6%)
M		
0	177 (94.7%)	178 (95.7%)
1	10 (5.3%)	8 (4.3%)
TNM stage		
I	123 (65.8%)	111 (59.7%)
II	10 (5.3%)	17 (9.1%)
III	45 (24.1%)	48 (25.8%)
IV	9 (4.8%)	10 (5.4%)
Surgery		
Nephron-sparing (open)	20 (10.7%)	23 (12.4%)
Nephron-sparing (laparoscopic)	0 (0%)	1 (0.5%)
Radical nephrectomy (open)	152 (81.3%)	146 (78.5%)
Radical nephrectomy (laparoscopic)	15 (8.0%)	16 (8.6%)

Data are median (IQR) or n (%). ECOG PS=Eastern Cooperative Oncology Group performance status

### Prognostic values

**Figure 2:**
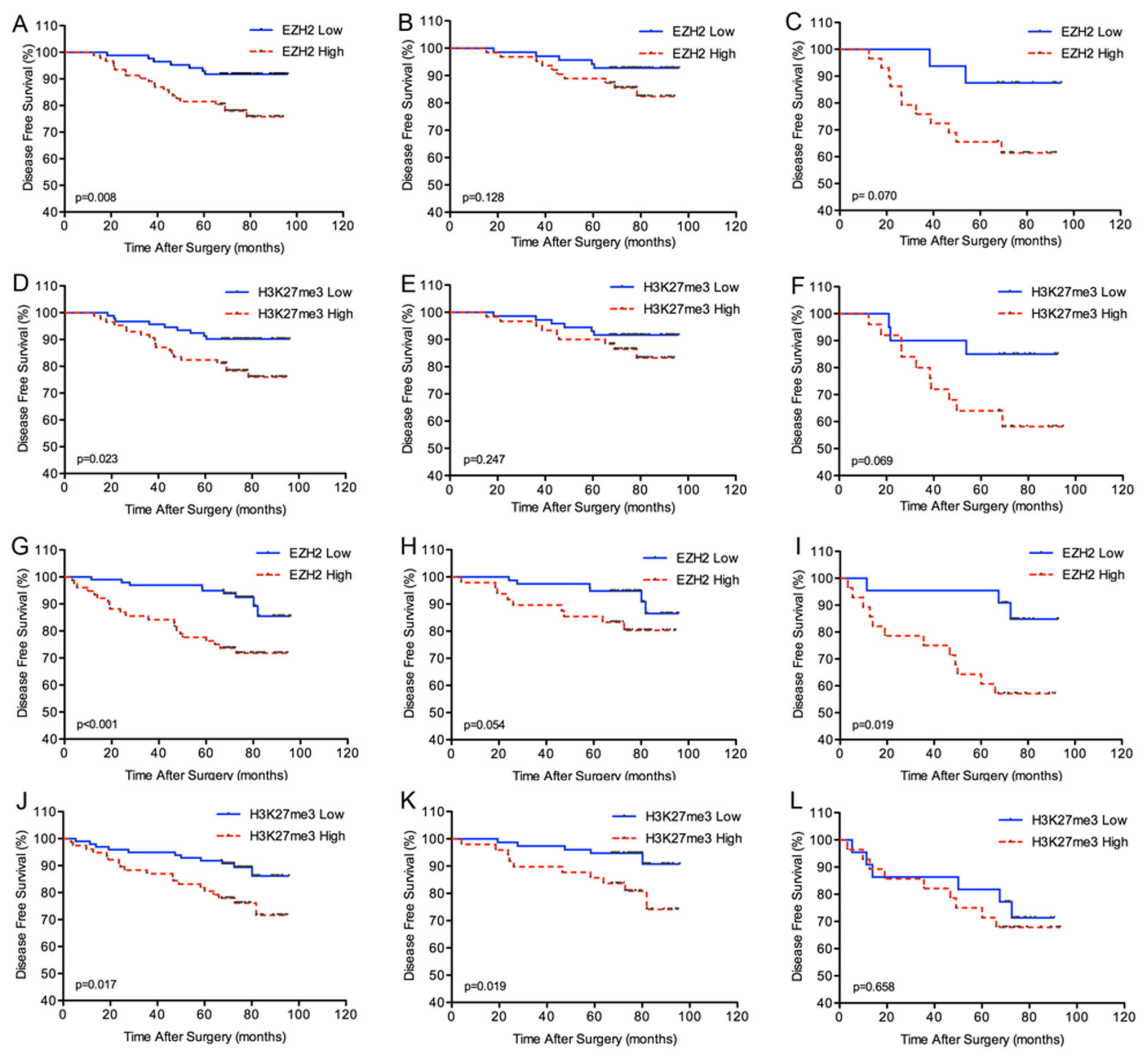
Kaplan-Meier analysis of disease-free survival (DFS) in renal cell carcinoma according to expression of the EZH2 or H3K27me3 score. (A), (D) all patients in the training set. (B), (E) patients with I+II stage disease in the training set. (C), (F) patients with III+IV stage disease in the training set. (G), (J) all patients in the validation set. (H), (K) patients with I+II stage disease in the validation set. (I), (L) patients with III+IV stage disease in the validation set.

**Figure 3:**
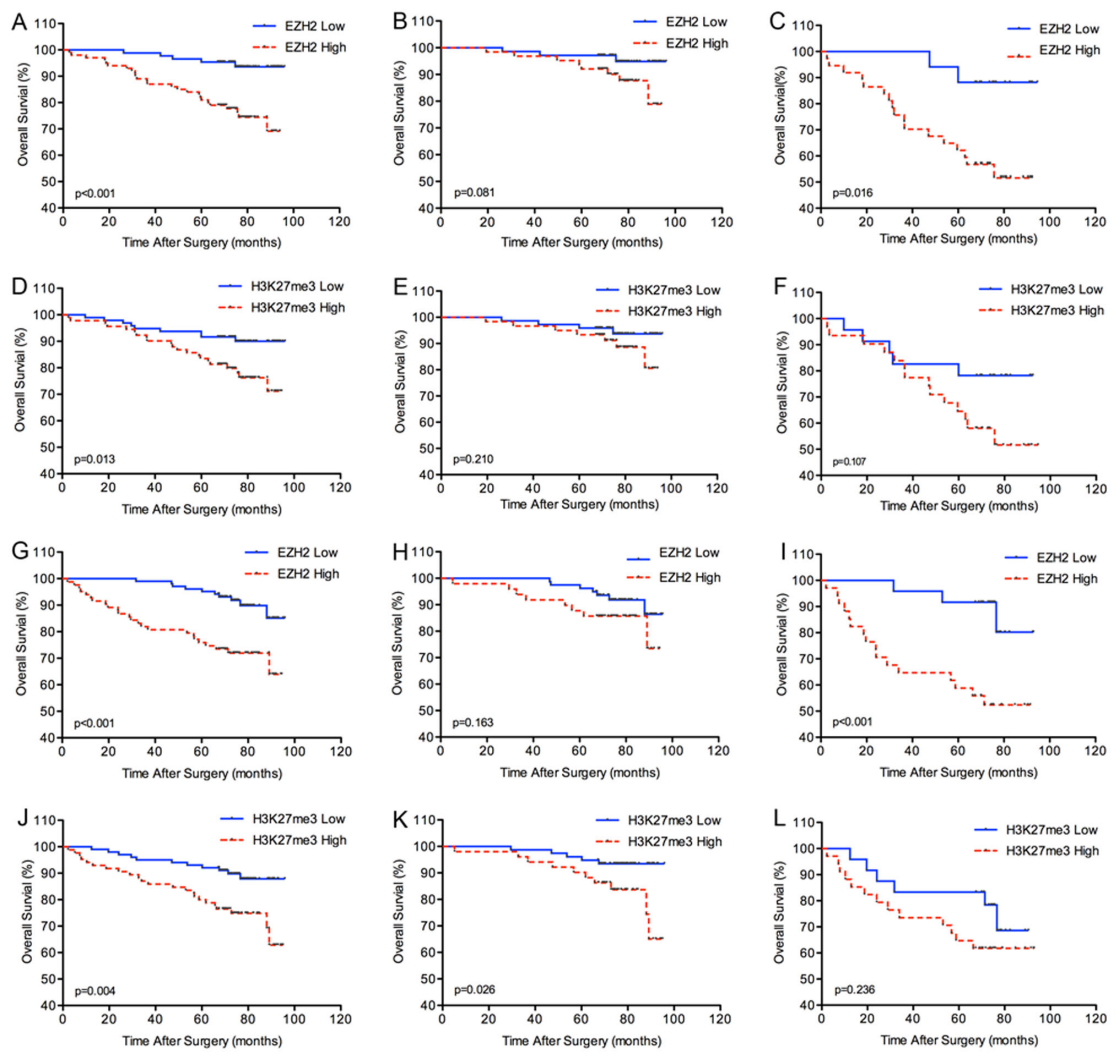
Kaplan-Meier analysis of overall survival (OS) in renal cell carcinoma according to expression of the EZH2 or H3K27me3 score. (A), (D) all patients in the training set. (B), (E) patients with I+II stage disease in the training set. (C), (F) patients with III+IV stage disease in the training set. (G), (J) all patients in the validation set. (H), (K) patients with I+II stage disease in the validation set. (I), (L) patients with III+IV stage disease in the validation set.

DFS was our first outcome of interest. Ten patients in the training set and 11 patients in the validation set were excluded for distant metastasis before surgery or death from other causes (table S4). In both sets, EZH2 or H3K27me3 high expression is associated with poor DFS (Figure 2). In univariate analysis, intratumoral EZH2 and H3K27me3 expression levels are significant predictor for DFS in training set (hazard ratio [HR] 3.005, 95%CI 1.28-7.07; p=0.012; and 2.444, 1.11-5.40; p=0.027, respectively) (table S5), and validation set (HR 3.501, 95%CI 1.60-7.65; p=0.002; and 2.394, 1.14-5.03; p=0.021, respectively) (table S6). In multivariate Cox regression analysis, only EZH2 expression (p=0.019, p=0.002, respectively), TNM stage (p=0.032, p=0.024, respectively), and ECOG PS (p=0.046, p=0.010, respectively) are independent prognostic factors for DFS in training and validation sets (table 2). In the validation set, patients with EZH2 high expression have a significantly higher probability of metastasis or recurrence in advanced clinical stage (p=0.019; Figure 2 I). Although the differences do not reach the significance using log-rank analysis in the training set, there is a similar tendency in the training set (Figure 2 C). The data showed that high EZH2 expression (p<0.001 and p<0.001, respectively) and H3K27me3 high level (p=0.013 and p=0.004, respectively) correlated with poor overall survival in training and validation sets using log-rank test analysis (Figure 3). Importantly, EZH2 high expression also associates with poor OS in patients with advanced clinical stage in training set and validation set (p=0.016 and p<0.001, respectively) (Figure 3). In agreement with previous studies, our data demonstrate that RCC patients with high ECOG PS, advanced Fuhrman grade, and advanced TNM clinical stage have a high risk of death, metastasis or recurrence using univariate analysis. In multivariate Cox regression analysis, EZH2 expression (p=0.004 and p=0.002, respectively) and TNM stage (p<0.001 and p=0.007, respectively) are independent prognostic factors for OS adjusted with age, ECOG PS, grade, and TNM stage in both sets (table 2).

**Table 2:** Multivariable Cox regression analysis of EZH2 and H3K27me3 expression and survival in training and validation sets.

	Training set			Validation set		
Variable	Hazard Ratio	95%CI	P	Hazard Ratio	95%CI	P
OS						
Age, years (≤55 v >55)	1.360	0.63-2.93	0.434	2.167	1.07-4.39	0.032
ECOG PS (0 v ≥ 1)	1.732	0.82-3.67	0.151	1.869	0.93-3.75	0.078
Fuhrman grade (1-2 v 3-4)	1.328	0.54-3.25	0.535	3.731	1.84-7.51	<0.001
TNM stage (I-II v III-IV)	4.516	2.08-9.80	<0.001	2.603	1.29-5.24	0.007
Intratumoral EZH2						
(Low v High)	4.106	1.56-10.82	0.004	3.266	1.54-6.93	0.002
Intratumoral H3K27me3						
(Low v High)	2.266	1.01-5.07	0.046	2.072	0.99-4.34	0.053
DFS						
Age, years (≤55 v >55)	1.335	0.62-2.89	0.464	2.701	1.25-5.84	0.012
ECOG PS (0 v ≥ 1)	2.197	1.02-4.75	0.046	2.575	1.25-5.31	0.010
Fuhrman grade (1-2 v 3-4)	1.796	0.74-4.39	0.199	3.991	1.85-8.61	<0.001
TNM stage (I-II v III-IV)	2.328	1.08-5.03	0.032	2.311	1.11-4.79	0.024
Intratumoral EZH2						
(Low v High)	2.801	1.19-6.62	0.019	3.626	1.61-8.15	0.002
Intratumoral H3K27me3						
(Low v High)	2.212	0.98-4.98	0.055	1.601	0.74-3.47	0.232

ECOG PS= Eastern Cooperative Oncology Group performance status； CI= confidence interval; OS= overall survival

DFS= disease free survival.

### Improved risk stratification model

**Figure 4:**
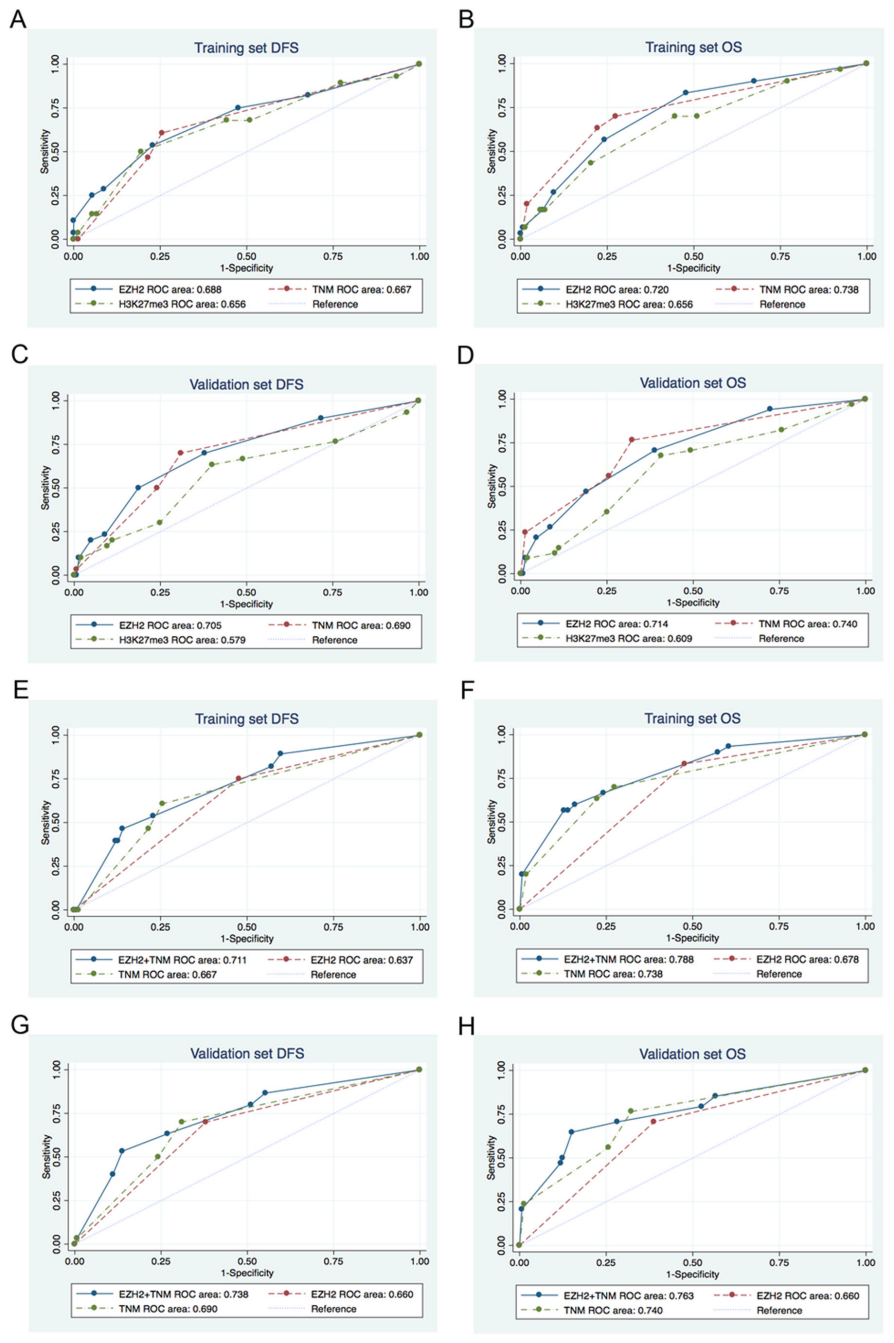
Comparisons of the receiver operating characteristic (ROC) curves for prediction of survival by the EZH2 score, TNM stage, and H3K27me3 score. (A) (E) DFS, (B) (F) OS in the training set. (C) (G) DFS, (D) (H) OS in the validation set. (A-D) the area under the ROC curves (AUROC) of EZH2 score versus the AUROC of TNM stage, or H3K27me3 score. (E-H) the AUROC of the combined EZH2 and TNM stage model versus the AUROC of the TNM stage or EZH2 expression alone model.

TNM stage is the most common used clinical prognosticator for RCC patients. When compared with TNM stage, the predictive value of the EZH2 expression is much the same using ROC analysis in both sets (all p>0.05) (Figure 4) (table 3) (table 4). To develop a more sensitive predictive tool, we created a new prognostic score model combining EZH2 expression and TNM stage which are two independent prognostic factors based on the both sets. H3K27me3 expression was excluded in the model because the p-value was greater than 0.051 to value prognosis using Logistic stepwise regression. Combination of EZH2 expression and TNM stage has a better prognostic value of DFS and OS than does TNM stage or EZH2 expression alone in training set (p=0.009, p<0.001, respectively) (Figure 4) (table 3) and validation set (p<0.001, p<0.001, respectively) (Figure 4) (table 4). 

**Table 3:** Comparisons of the receiver operating characteristic (ROC) curves for prediction of survival in training set.

	Training set DFS			Training set OS		
Variable	AUROC	95%CI	p	AUROC	95%CI	p
EZH2	0.688	0.57-0.80	0.850	0.720	0.62-0.82	0.404
TNM	0.667	0.57-0.77		0.738	0.64-0.83	
H3K27me3	0.656	0.54-0.77		0.656	0.55-0.76	
EZH2+TNM	0.711	0.61-0.81	0.009	0.788	0.70-0.88	<0.001
EZH2	0.637	0.55-0.73		0.678	0.60-0.76	
TNM	0.667	0.57-0.77		0.738	0.64-0.83	

AUROC= area under the receiver operating characteristic curves; CI= confidence interval; OS= overall survival; DFS= disease free survival.

**Table 4:** Comparisons of the receiver operating characteristic (ROC) curves for prediction of survival in validation set.

	Validation set DFS			Validation set OS		
Variable	AUROC	95%CI	p	AUROC	95%CI	p
EZH2	0.705	0.60-0.81	0.097	0.714	0.62-0.81	0.116
TNM	0.690	0.60-0.79		0.740	0.65-0.83	
H3K27me3	0.579	0.46-0.70		0.609	0.50-0.71	
EZH2+TNM	0.738	0.64-0.84	<0.001	0.763	0.67-0.86	<0.001
EZH2	0.660	0.57-0.75		0.660	0.57-0.75	
TNM	0.690	0.60-0.79		0.740	0.65-0.83	

AUROC= area under the receiver operating characteristic; CI= confidence interval; OS= overall survival; DFS= disease free survival.

## Discussion

EZH2, as a histone methyltransferase, catalyzes the trimethylation of the lysine 27 of histone H3 (H3K27me3). Our findings demonstrate that EZH2 is an independent poor prognostic factor for RCC patients undergoing nephrectomy. To our knowledge, it is the first study analysis the prognostic significance of EZH2 and H3K27me3 expression in RCC. Pathologic stages have proved to be the single most important prognostic factor for RCC. However, same cancer stage might still have diverse clinical outcomes. In this study, EZH2 expression is found to be predictive of survival of RCC patients independently. The prognostic value of TNM stage is further improved when EZH2 levels and TNM stage are combined as a predictive model. Moreover, EZH2 expression has a similar outcome predictive ability to TNM stage in ROC analysis and has good discriminatory power as a supplementary risk factor in patients with late-stage RCC. Evaluating EZH2 and H3K27me3 expression can be a clinically applicable procedure to discriminate patients with different prognosis. It could also lead to a more personalized treatment for RCC patients after curative surgery. 

Traditionally, cancer has been acknowledged as a genetic disease that is driven by the sequence of oncogenes or tumor suppressor genes alteration. However, epigenetic changes including histone modification, DNA methylation, and miroRNA regulation, are increasingly evident as a tumor developmental factor [19-21]. Once the DNA sequence is changed by mutation, it is hard to restore. While, epigenetic changes can be restored by inhibitors. PRC2 group as a transcriptional repressor plays a vital role in the control of cellular proliferation and neoplastic development. PRC2 nature activity is to methylate H3K27. The main form of H3K27 methylation is adding up to three methyl groups to the Lys residue for several silencing phenomena including homeotic-gene silencing, X inactivation and genomic imprinting [22]. PRC2 has four core subunits. However, EZH2 and SUZ12 play the opposite role. The active site in humans is EZH2 with the SET domain of the catalytic subunit [23]. In this study, we exclusively focused on EZH2 proteins. In many tumor types, such as prostate, breast, bladder, and gastric cancer, EZH2 levels are elevated in cancer tissues and associated with poor prognosis. Previous studies suggested that Ki-67 could be a predictive factor in RCC [24]. In a recent study, EZH2 expression was associated with Ki-67 in endometrium, prostate, and breast cancer [12]. Although, Stefan Hinz and colleagues showed that high EZH2 level indicated a less aggressive phenotype with a favorable prognosis in RCC patients [25]. Our present study indicates that EZH2 is an independent unfavorable prognostic marker and can predict poor outcome in late-stage RCC. These contrary findings might result from different genetic background and pathological feature of RCC patients. 

Our data showed that EZH2 has a better predictive outcome than H3K27me3 not only in late stage patients but also in all patients. Table S7 displays that the expression of EZH2 is positively correlative with H3K27me3. However, they are not completely consistent in Figure 1 I. The following reasons might be responsible for the discrepancy. First, it was reported that EZH2 was indispensable in DNA methyltransferases (DNMTs) binding and CpG methylation of target genes [26]. Second, EZH2 mediated transcriptional silencing can be blocked by the histone deacetylase (HDAC) inhibitor TSA, which suggested that the silencing enzymes played a synergistic role in vivo [27]. Third, the level of H3K27me3 can also be regulated by UTX and JMJD3, which are H3K27 demethylases [28]. The underlying molecular roles of EZH2 in RCC progression remain far from fully understood and merit further investigation.

Although several integrated staging systems (ISSs) have evolved and improved for prediction of prognosis and outcome for RCC, TNM staging system is still the most used clinical prognosticator and the essential guide to the evaluation of therapeutic options but is done on the basis of anatomical information [3,29]. Metastatic RCC (mRCC) is highly refractory to radiation and chemotherapy. Kinase inhibitors have a response rate of approximately 15% with severe side effects and toxicity [30]. The level of EZH2 provides the biological characteristics different from TNM staging system. Thus, it can be used to distinguish the outcome of late-stage RCC. EZH2 plays the major role in histone methylation, which can be reversed by inhibitors. 

## Conclusion

Our results prospectively suggest that high intratumoral EZH2 expression independently predicts poor postoperative outcome of RCC patients. Integration of intratumoral EZH2 expression levels into current TNM staging systems might improve prognostic value to patient survival and contribute to personalized therapy.

## Supporting Information

Table S1 **New variable predicted probability (p) for RCC survival.**
(DOCX)

Table S2 **Clinical characteristics of patients according to the EZH2 and H3K27me3 expression in the training set (n=187).**
(DOCX)

Table S3 **Clinical characteristics of patients according to the EZH2 and H3K27me3 expression in the validation set (n=186).**
(DOCX)

Table S4 **Patients were excluded form DFS analysis.**
(DOCX)

Table S5 **Training set univariate analyses of factors associated with overall survival and disease free survival.**
(DOCX)

Table S6 **Validation set univariate analyses of factors associated with overall survival and disease free survival.**
(DOCX)

Table S7 **EZH2 expression positively correlates with H3K27me3 in both sets.**
(DOCX)
