# Supplementary material for: Prognostic Value of EZH2 Expression and Activity in Renal Cell Carcinoma: A Prospective Study
Source: PLoS One. 2013 Nov 27;8(11):e81484. doi: 10.1371/journal.pone.0081484 (PMC3842247; doi:10.1371/journal.pone.0081484)
Supplement: Table S1 — New variable predicted probability (p) for RCC survival. (DOCX) [file pone.0081484.s001.docx]

**Table S1: New variable predicted probability (p) for RCC survival**

|  | **Regression equations** |
| --- | --- |
| Training set DFS | Ln (p/1-p)=-3.403+0.602TNM+1.086EZH2 |
| Training set OS | Ln (p/1-p)=-4.347+0.853TNM+1.477EZH2 |
| Validation set DFS | Ln (p/1-p)=-3.452+0.664TNM+1.159EZH2 |
| Validation set OS | Ln (p/1-p)=-3.745+0.819TNM+1.035EZH2 |
