# Supplementary material for: Prognostic Value of EZH2 Expression and Activity in Renal Cell Carcinoma: A Prospective Study
Source: PLoS One. 2013 Nov 27;8(11):e81484. doi: 10.1371/journal.pone.0081484 (PMC3842247; doi:10.1371/journal.pone.0081484)
Supplement: Table S2 — Clinical characteristics of patients according to the EZH2 and H3K27me3 expression in the training set (n=187). (DOCX) [file pone.0081484.s002.docx]

**Table S2: Clinical characteristics of patients according to the EZH2 and H3K27me3 expression in the training set (n=187)**

|  | EZH2 positive | | | H3K27me3 positive | | |
| --- | --- | --- | --- | --- | --- | --- |
| Characteristic | Low | High | p | Low | High | p |
| ALL patients | 87 (46.5%) | 100 (53.5%) |  | 96 (51.3%) | 91 (48.7%) |  |
| Age, years |  |  | 0.493† |  |  | 0.707† |
| ≤55 | 47 (25.1%) | 49 (26.2%) |  | 48 (25.7%) | 48 (25.7%) |  |
| >55 | 40 (21.4%) | 51 (27.3%) |  | 48 (25.7%) | 43 (22.9%) |  |
| Sex |  |  | 0.057† |  |  | 0.160† |
| Female | 30 (16.0%) | 22 (11.8%) |  | 31 (16.6%) | 21 (11.2%) |  |
| Male | 57 (30.5%) | 78 (41.7%) |  | 65 (34.8%) | 70 (37.4%) |  |
| Histology |  |  | 0.166† |  |  | 0.509† |
| Clear cell | 78 (41.7%) | 95 (50.8%) |  | 90 (48.1%) | 83 (44.4%) |  |
| Others | 9 (4.8%) | 5 (2.7%) |  | 6 (3.2%) | 8 (4.3%) |  |
| ECOG PS |  |  | 0.177† |  |  | 0.472† |
| 0 | 70 (37.4%) | 72 (38.5%) |  | 75 (40.1%) | 67 (35.9%) |  |
| ≥1 | 17 (9.1%) | 28 (15.0%) |  | 21 (11.2%) | 24 (12.8%) |  |
| Fuhrman grade |  |  | 0.698‡ |  |  | 0.102‡ |
| 1 | 13 (7.0%) | 16 (8.6%) |  | 16 (8.6%) | 13 (7.0%) |  |
| 2 | 64 (34.2%) | 69 (36.9%) |  | 72 (38.5%) | 61 (32.6%) |  |
| 3 | 9 (4.8%) | 13 (7.0%) |  | 7 (3.8%) | 15 (8.0%) |  |
| 4 | 1 (0.5%) | 2 (1.0 %) |  | 1 (0.5%) | 2 (1.0%) |  |
| T classification |  |  | 0.079‡ |  |  | 0.324‡ |
| T1 | 64 (34.2%) | 62 (33.2%) |  | 66 (35.3%) | 60 (32.1%) |  |
| T2 | 6 (3.2%) | 6 (3.2%) |  | 9 (4.8%) | 3 (1.6%) |  |
| T3 | 16 (8.6%) | 32 (17.1%) |  | 21 (11.2%) | 27 (14.5%) |  |
| T4 | 1 (0.5%) | 0 (0%) |  | 0 (0%) | 1 (0.5%) |  |
| Distant metastasis |  |  | 0.078† |  |  | 0.144† |
| No | 85 (45.4%) | 92 (49.2%) |  | 93 (49.7%) | 84 (44.9%) |  |
| Yes | 2 (1.1%) | 8 (4.3%) |  | 3 (1.6%) | 7 (3.8%) |  |
| TNM stage |  |  | 0.012‡ |  |  | 0.228‡ |
| I | 64 (34.2%) | 59 (31.6%) |  | 65 (34.7%) | 58 (31.0%) |  |
| II | 6 (3.2%) | 4 (2.2%) |  | 8 (4.3%) | 2 (1.1%) |  |
| III | 15 (8.0%) | 30 (16.0%) |  | 20 (10.7%) | 25 (13.4%) |  |
| IV | 2 (1.1%) | 7 (3.7%) |  | 3 (1.6%) | 6 (3.2%) |  |

Data are n (%). †χ^2^ test or Fisher’s exact test. ‡Cochran-Mantel-Haenszel χ^2^ test. ECOG PS=Eastern Cooperative Oncology Group performance status
