# Supplementary material for: Prognostic Value of EZH2 Expression and Activity in Renal Cell Carcinoma: A Prospective Study
Source: PLoS One. 2013 Nov 27;8(11):e81484. doi: 10.1371/journal.pone.0081484 (PMC3842247; doi:10.1371/journal.pone.0081484)
Supplement: Table S3 — Clinical characteristics of patients according to the EZH2 and H3K27me3 expression in the validation set (n=186). (DOCX) [file pone.0081484.s003.docx]

**Table S3: Clinical characteristics of patients according to the EZH2 and H3K27me3 expression in the validation set (n=186)**

|  | EZH2 positive | | | H3K27me3 positive | | |
| --- | --- | --- | --- | --- | --- | --- |
| Characteristic | Low | High | p | Low | High | p |
| ALL patients | 103 (55.4%) | 83 (44.6%) |  | 101 (54.3%) | 85 (45.7%) |  |
| Age, years |  |  | 0.958† |  |  | 0.211† |
| ≤55 | 55 (29.6%) | 44 (23.7%) |  | 58 (31.2%) | 41 (22.0%) |  |
| >55 | 48 (25.8%) | 39 (20.9%) |  | 43 (23.1%) | 44 (23.7%) |  |
| Sex |  |  | 0.602† |  |  | 0.298† |
| Female | 36 (19.4%) | 26 (14.0%) |  | 37 (19.9%) | 25 (13.4%) |  |
| Male | 67 (36.0%) | 57 (30.6%) |  | 64 (34.4%) | 60 (32.3%) |  |
| Histology |  |  | 0.832† |  |  | 0.366† |
| Clear cell | 94 (50.6%) | 75 (40.3%) |  | 90 (48.4%) | 79 (42.5%) |  |
| Others | 9 (4.8%) | 8 (4.3%) |  | 11 (5.9%) | 6 (3.2%) |  |
| ECOG PS |  |  | 0.657† |  |  | 0.180† |
| 0 | 81 (43.5%) | 63 (33.9%) |  | 82 (44.1%) | 62 (33.3%) |  |
| ≥1 | 22 (11.8%) | 20 (10.8%) |  | 19 (10.2%) | 23 (12.4%) |  |
| Fuhrman grade |  |  | 0.077‡ |  |  | 0.047‡ |
| 1 | 14 (7.5%) | 7 (3.7%) |  | 14 (7.5%) | 7 (3.8%) |  |
| 2 | 74 (39.8%) | 58 (31.2%) |  | 73 (39.3%) | 59 (31.7%) |  |
| 3 | 15 (8.1%) | 16 (8.6%) |  | 14 (7.5%) | 17 (9.1%) |  |
| 4 | 0 (0%) | 2 (1.1%) |  | 0 (0%) | 2 (1.1%) |  |
| T classification |  |  | 0.011‡ |  |  | 0.008‡ |
| T1 | 71 (38.2%) | 44 (23.7%) |  | 71 (38.2%) | 44 (23.7%) |  |
| T2 | 10 (5.4%) | 9 (4.8%) |  | 8 (4.3%) | 11 (5.9%) |  |
| T3 | 22 (11.8%) | 27 (14.5%) |  | 22 (11.8%) | 27 (14.5%) |  |
| T4 | 0 (0%) | 3 (1.6%) |  | 0 (0%) | 3 (1.6%) |  |
| Distant metastasis |  |  | 0.080† |  |  | 0.090† |
| No | 101 (54.3%) | 77 (41.4%) |  | 99 (53.2%) | 79 (42.5%) |  |
| Yes | 2 (1.1%) | 6 (3.2%) |  | 2 (1.1%) | 6 (3.2%) |  |
| TNM stage |  |  | 0.002‡ |  |  | 0.003‡ |
| I | 71 (38.2%) | 40 (21.5%) |  | 70 (37.6%) | 41 (22.0%) |  |
| II | 8 (4.3%) | 9 (4.8%) |  | 7 (3.8%) | 10 (5.4%) |  |
| III | 22 (11.8%) | 26 (14.0%) |  | 22 (11.8%) | 26 (14.0%) |  |
| IV | 2 (1.1%) | 8 (4.3%) |  | 2 (1.1%) | 8 (4.3%) |  |

Data are n (%). †χ^2^ test or Fisher’s exact test. ‡Cochran-Mantel-Haenszel χ^2^ test. ECOG PS=Eastern Cooperative Oncology Group performance status
