# Supplementary material for: Prognostic Value of EZH2 Expression and Activity in Renal Cell Carcinoma: A Prospective Study
Source: PLoS One. 2013 Nov 27;8(11):e81484. doi: 10.1371/journal.pone.0081484 (PMC3842247; doi:10.1371/journal.pone.0081484)
Supplement: Table S4 — Patients were excluded form DFS analysis. (DOCX) [file pone.0081484.s004.docx]

**Table S4: Patients were excluded form DFS analysis**

| **Number** | **Reason** |
| --- | --- |
| Training set |  |
| 1 | Primary lung cancer after surgery |
| 2 | Lung metastases at diagnosis |
| 3 | Lung and rectum metastases at diagnosis |
| 4 | Right ilium metastases at diagnosis |
| 5 | Primary bladder cancer after surgery |
| 6 | Lung metastases at diagnosis |
| 7 | Retroperitoneal lymph nodes and Lateral abdominal wall metastases at diagnosis |
| 8 | Lung metastases at diagnosis |
| 9 | Right abdominal skin metastases at diagnosis |
| 10 | Lung metastases at diagnosis |
| Validation set |  |
| 1 | Lung metastases at diagnosis |
| 2 | Bone metastases at diagnosis |
| 3 | Retroperitoneal lymph nodes metastases at diagnosis |
| 4 | Lung metastases at diagnosis |
| 5 | Lung metastases at diagnosis |
| 6 | Died of myocardial infarction |
| 7 | Primary pancreatic carcinoma after surgery |
| 8 | Died of accident |
| 9 | The cause of death is unknown |
| 10 | Tail of pancreas metastases at diagnosis |
| 11 | Lung metastases at diagnosis |
