# Supplementary material for: Prognostic Value of EZH2 Expression and Activity in Renal Cell Carcinoma: A Prospective Study
Source: PLoS One. 2013 Nov 27;8(11):e81484. doi: 10.1371/journal.pone.0081484 (PMC3842247; doi:10.1371/journal.pone.0081484)
Supplement: Table S5 — Training set univariate analyses of factors associated with overall survival and disease free survival. (DOCX) [file pone.0081484.s005.docx]

**Table S5: Training set univariate analyses of factors associated with overall survival and disease free survival**

|  | OS | | | DFS | | |
| --- | --- | --- | --- | --- | --- | --- |
| Variable | Hazard Ratio | 95%CI | p | Hazard Ratio | 95%CI | p |
| Age, years (≤55 v >55） | 1.699 | 0.82-3.53 | 0.155 | 1.579 | 0.75-3.34 | 0.232 |
| Sex (Female v Male） | 1.063 | 0.47-2.39 | 0.882 | 0.936 | 0.41-2.13 | 0.874 |
| Histology |  |  |  |  |  |  |
| **(**ccRCC v others) | 2.334 | 0.32-17.15 | 0.405 | 2.248 | 0.31-16.55 | 0.427 |
| ECOG PS (0 v ≥1) | 2.308 | 1.11-4.79 | 0.025 | 2.709 | 1.28-5.73 | 0.009 |
| Fuhrman grade **(**1-2 v 3-4) | 2.094 | 0.89-4.88 | 0.087 | 2.427 | 1.03-5.71 | 0.042 |
| TNM stage (I-II v III-IV） | 5.301 | 2.52-11.16 | <0.001 | 2.972 | 1.39-6.15 | 0.005 |
| Intratumoral EZH2 |  |  |  |  |  |  |
| **(**Low v High) | 4.883 | 1.87-12.76 | 0.001 | 3.005 | 1.28-7.07 | 0.012 |
| Intratumoral H3K27me3 |  |  |  |  |  |  |
| (Low v High) | 2.605 | 1.19-5.69 | 0.016 | 2.444 | 1.11-5.40 | 0.027 |

ECOG PS= Eastern Cooperative Oncology Group performance status； CI= confidence interval; OS= overall survival

DFS= disease free survival.
