# Supplementary material for: Prognostic Value of EZH2 Expression and Activity in Renal Cell Carcinoma: A Prospective Study
Source: PLoS One. 2013 Nov 27;8(11):e81484. doi: 10.1371/journal.pone.0081484 (PMC3842247; doi:10.1371/journal.pone.0081484)
Supplement: Table S6 — Validation set univariate analyses of factors associated with overall survival and disease free survival. (DOCX) [file pone.0081484.s006.docx]

**Table S6: Validation set univariate analyses of factors associated with overall survival and disease free survival**

|  | OS | | | DFS | | |
| --- | --- | --- | --- | --- | --- | --- |
| Variable | Hazard Ratio | 95%CI | p | Hazard Ratio | 95%CI | p |
| Age, years (≤55 v >55） | 1.967 | 0.98-3.93 | 0.055 | 2.301 | 1.09-4.84 | 0.028 |
| Sex (Female v Male） | 1.433 | 0.67-3.07 | 0.354 | 1.199 | 0.55-2.62 | 0.650 |
| Histology |  |  |  |  |  |  |
| **(**ccRCC v others) | 0.486 | 0.19-1.27 | 0.139 | 0.558 | 0.19-1.61 | 0.281 |
| ECOG PS (0 v ≥1) | 2.156 | 1.08-4.31 | 0.030 | 3.172 | 1.55-6.51 | 0.002 |
| Fuhrman grade **(**1-2 v 3-4) | 4.345 | 2.21-8.56 | <0.001 | 4.093 | 1.97-8.51 | <0.001 |
| TNM stage (I-II v III-IV） | 3.456 | 1.75-6.82 | <0.001 | 2.879 | 1.41-5.90 | 0.004 |
| Intratumoral EZH2 |  |  |  |  |  |  |
| **(**Low v High) | 3.526 | 1.69-7.38 | 0.001 | 3.501 | 1.60-7.65 | 0.002 |
| Intratumoral H3K27me3 |  |  |  |  |  |  |
| (Low v High) | 2.749 | 1.34-5.64 | 0.006 | 2.394 | 1.14-5.03 | 0.021 |

ECOG PS= Eastern Cooperative Oncology Group performance status； CI= confidence interval; OS= overall survival

DFS= disease free survival.
