# Supplementary material for: Prognostic Value of EZH2 Expression and Activity in Renal Cell Carcinoma: A Prospective Study
Source: PLoS One. 2013 Nov 27;8(11):e81484. doi: 10.1371/journal.pone.0081484 (PMC3842247; doi:10.1371/journal.pone.0081484)
Supplement: Table S7 — EZH2 expression positively correlates with H3K27me3 in both sets. (DOCX) [file pone.0081484.s007.docx]

**Table S7: EZH2 expression positively correlates with H3K27me3 in both sets.**

|  | Training set | | Validation set | |
| --- | --- | --- | --- | --- |
|  | EZH2 | | EZH2 | |
| H3K27me3 | Low | High | Low | High |
| Low | 62 | 34 | 75 | 26 |
| High | 25 | 66 | 28 | 57 |
| r | 0.372 | | 0.414 | |
| p | <0.001 | | <0.001 | |

r, Spearman correlation coefficient.
